# Seawater cultivation of freshwater cyanobacterium *Synechocystis* sp. PCC 6803 drastically alters amino acid composition and glycogen metabolism

**DOI:** 10.3389/fmicb.2015.00326

**Published:** 2015-04-22

**Authors:** Hiroko Iijima, Yuka Nakaya, Ayuko Kuwahara, Masami Yokota Hirai, Takashi Osanai

**Affiliations:** ^1^RIKEN Center for Sustainable Resource ScienceYokohama, Japan; ^2^Advanced Low Carbon Technology Research and Development Program (ALCA), Japan Science and Technology AgencyKawaguchi, Japan; ^3^School of Agriculture, Meiji UniversityTokyo, Japan

**Keywords:** amino acids, artificial seawater, cyanobacteria, natural seawater, *Synechocystis*

## Abstract

Water use assessment is important for bioproduction using cyanobacteria. For eco-friendly reasons, seawater should preferably be used for cyanobacteria cultivation instead of freshwater. In this study, we demonstrated that the freshwater unicellular cyanobacterium *Synechocystis* sp. PCC 6803 could be grown in a medium based on seawater. The *Synechocystis* wild-type strain grew well in an artificial seawater (ASW) medium supplemented with nitrogen and phosphorus sources. The addition of HEPES buffer improved cell growth overall, although the growth in ASW medium was inferior to that in the synthetic BG-11 medium. The levels of proteins involved in sugar metabolism changed depending on the culture conditions. The biosynthesis of several amino acids including aspartate, glutamine, glycine, proline, ornithine, and lysine, was highly up-regulated by cultivation in ASW. Two types of natural seawater (NSW) were also made available for the cultivation of *Synechocystis* cells, with supplementation of both nitrogen and phosphorus sources. These results revealed the potential use of seawater for the cultivation of freshwater cyanobacteria, which would help to reduce freshwater consumption during biorefinery using cyanobacteria.

## Introduction

The use of oxygenic photosynthetic prokaryotes as cell factories to directly convert CO_2_ and water into compounds of interests by light is required for the sustainable development of the society (Branco dos Santos et al., 2014). Cyanobacteria are a group of photosynthetic bacteria that undergo oxygenic photosynthesis and fix CO_2_ via the Calvin-Benson cycle. The freshwater cyanobacterium *Synechocystis* sp. PCC 6803 (hereafter *Synechocystis* 6803) is the most widely studied species among cyanobacteria, owing to their abilities of natural transformation and fast growth (Yu et al., 2013). The production of valuable products such as alcohols, alkanes, bioplastics, fatty acids, and hydrogen has been achieved by genetically engineering *Synechocystis* 6803, indicating the potential use of this bacterium as a biocatalyst (Savakis and Hellingwerf, 2014). Recent advances in the metabolome analysis of *Synechocystis* 6803 support this idea to promote the metabolic engineering of cyanobacteria (Osanai et al., 2011, 2014b).

A life cycle assessment concluded that algal cultivation requires a much larger amount of freshwater than do conventional crops, leading to high environmental impacts with algal biorefineries (Clarens et al., 2010). The utilization of seawater and/or wastewater as alternatives would reduce the freshwater requirement of algae and cyanobacteria cultivation. Another life cycle assessment indicated, however, that wastewater-based microalgal bioproduction also has large environmental impacts, due to the need to process the wastewater for algae growth (Mu et al., 2014). Thus, growth in seawater is preferable for bioproduction using microalgae and cyanobacteria (Savakis and Hellingwerf, 2014). However, there are not many studies on the use of seawater for cultivating model cyanobacteria except marine species.

*Synechocystis* 6803 is known to be a freshwater cyanobacterium that is able to grow under high salt (NaCl) conditions and a near-costal area in biofilms (Reed et al., 1985; Gram et al., 2002). An increase in NaCl induces the influx of Na^+^ and Cl^−^ into the cells. This activates the Na^+^/H^+^ antiporter, which decreases the intracellular Na^+^ concentration, and results in an increase in the K^+^ concentration to compensate (Reed et al., 1985; Hagemann, 2011). *Synechocystis* 6803 cells then accumulate compatible solutes such as glucosylglycerol and sucrose to acclimate to the high salt conditions (Reed et al., 1985; Hagemann, 2011). These compatible solutes could be imported directly from the surrounding environment into the *Synechocystis* 6803 cells (Mikkat et al., 1996, 1997). The expression of genes encoding ribosomal proteins, chaperones, and enzymes for glucosylglycerol synthesis are up-regulated by 500 mM NaCl within 30 min, while the gene expression levels related to phycobilisomes, Photosystem I subunits, and desaturases are decreased (Kanesaki et al., 2002; Marin et al., 2004). Proteomic analysis has also demonstrated increased protein levels of glucosylglycerol biosynthesis, glucosylglycerol-phosphate synthase (GgpS) and glucosylglycerol-phosphate phosphatase (GgpP or StpA), chaperones (GroEL1, DnaK2, and GrpE), elongation factors, and general stress proteins (Fulda et al., 2006). Nevertheless, the relationship between salt conditions and metabolite levels in primary metabolism has not been studied in detail. Recent metabolomic analyses have indicated that the growth of *Synechocystis* 6803 is closely associated with sugar and amino acid metabolism (Osanai et al., 2014a,d).

In this study, we revealed that *Synechocystis* 6803 cells could grow in a seawater-based medium supplemented with nitrogen and phosphorus sources, and that the addition of HEPES buffer improved cell proliferation. Several amino acids were accumulated in high amounts in the artificial seawater (ASW) medium, revealing the altered primary metabolism that occurs during seawater cultivation.

## Materials and methods

### Bacterial strains and culture conditions

The glucose-tolerant (GT) strain of *Synechocystis* sp. PCC 6803, isolated by Williams (1988) and the GT-I strain among GT strains was used in this study (Kanesaki et al., 2012). For preculture, the GT cells were grown in modified BG-11 medium, which is BG-11_0_ liquid medium containing 5 mM NH_4_Cl [buffered with 20 mM 4-(2-hydroxyethyl)piperazine-1-ethanesulfonic acid (HEPES)-KOH, pH 7.8] (Rippka, 1988). Marine Art SF-1 (Osaka Yakken, Osaka, Japan) was used as the ASW medium and contained 22.1 g/L NaCl, 9.9 g/L MgCl_2_•6H_2_O, 1.5 g/L CaCl_2_•2H_2_O, 3.9 g/L Na_2_SO_4_, 0.61 g/L KCl, 0.19 g/L NaHCO_3_, 96 mg/L KBr, 78 mg/L Na_2_B_4_O_7_•10H_2_O, 13 mg/L SrCl_2_, 3 mg/L NaF, 1 mg/L LiCl, 81 μg/L KI, 0.6 μg/L MnCl_2_•4H_2_O, 2 μg/L CoCl_2_•6H_2_O, 8 μg/L AlCl_3_•6H_2_O, 5 μg/L FeCl_3_•6H_2_O, 2 μg/L Na_2_WO_4_•2H_2_O, and 18 μg/L (NH_4_)_6_Mo_7_O_24_•4H_2_O. Two natural seawater samples were used as cultivation media, designated as NSWS (Shimano-Tennensui, seawater around Ohshima, south island of Tokyo; provided by NIHON AQUARIUM, Tokyo, Japan) and NSWN (NAGEME10, seawater around Izu Peninsula; provided by Bluelab Co. Ltd., Shizuoka, Japan). Liquid cultures were bubbled with 1% (v/v) CO_2_ in air and incubated at 30°C under continuous white light (~50–70 μmol photons m^−2^·s^−1^). Cell growth and densities were measured at OD_730_ with a Hitachi U-3310 spectrophotometer (Hitachi High-Tech., Tokyo, Japan). The pH values of the media were measured with the pH meter F-52 (HORIBA, Kyoto, Japan), using the supernatant of the cultures after centrifugation (5800 × *g* for 2 min).

### Glycogen measurement

Glycogen was quantified by the Biotechnology Center of Akita Prefectural University, Japan. Cells cultivated for 3 days were concentrated to an OD_730_ value of 6.0 in 1 mL of methanol, mixed for 10 min using a vortex mixer and then pelleted by centrifugation. The supernatant was transferred to a 1.5-mL tube and dried at 65°C. Cells were resuspended in 1 mL distilled water and incubated at 100°C for 40 min. 200 μL of cell suspension was transferred to a 1.5-mL tube and the resultant monosaccharides were enzymatically degraded with 100 μL glucoamylase solution (75 U/mL), and the resultant glucose was measured by estimating the changes in OD_340_ during hexokinase and glucose-6-phosphate dehydrogenase (G6PD) reactions.

### Chlorophyll measurement

Chlorophyll contents of the cells grown for 3 days were measured using a methanol extraction method (Grimme and Boardman, 1972). 1 mL of cell culture was transferred to a 1.5-mL tube and centrifuged (20500 × *g* for 2 min) and the supernatant was removed. Cells were suspended in 1 mL methanol and vortexed for 5 min. After placing 5 min, the cell suspensions were centrifuged (20500 × *g* for 2 min) and OD_665_ of the supernatants were quantified with a Hitachi U-3310 spectrophotometer.

### Quantification of intracellular protein levels

5 mL of cell culture was transferred to a 15-mL tube after cultivation for 3 days. The cell cultures were centrifuged (9000 × *g* for 2 min) and the supernatant was removed. Cells were suspended in 500 μL PBS-T (3.2 mM Na_2_HPO_4_, 0.5 mM KH_2_PO_4_, 1.3 mM KCl, 135 mM NaCl, and 0.05% Tween-20, pH 7.4) supplemented with the protease inhibitor Complete Mini (Roche Diagnostics, Rotkreuz, Switzerland; one tablet/30 mL and disrupted by sonication using VC-750 instrument (EYELA, Tokyo, Japan). After centrifugation at 9000 × *g* for 5 min at 4°C, the protein concentration in the soluble fraction was measured using BCA Protein Assay Reagent (Thermo Scientific Hudson, NH, USA) and bovine serum albumin as a standard.

### Antisera production and immunoblotting

We previously produced antisera against isoamylases [GlgX(slr0237), GlgX(slr1857)], glycogen phosphorylases [GlgP(sll1356), GlgP(slr1367)], glyceraldehyde-3-phosphate dehydrogenase (Gap2), G6PD, 6-phosphogluconate dehydrogenase (6PGD), and an RNA polymerase sigma factor SigE (Azuma et al., 2011; Osanai et al., 2011, 2013b). Antiserum against transaldolase (Tal) was produced by Sigma-Aldrich (St. Louis, Missouri, USA) using synthetic peptide NH_2_-CHAYDLDGDGFITREEWAG-COOH. Antiserum against fructose-1,6-bisphosphate aldolase (FbaI, slr0943) was commercially produced by Tampaku Seisei Kogyo (Gunma, Japan). To produce anti-FbaI, glutathione S-transferase (GST)-fused FbaI proteins were first purified. The *fbaI* DNA fragment was amplified by PCR with KOD polymerase (Toyobo, Osaka, Japan) and the specific primers; 5′-ATGGGATCCCCATGATGACTCTCGAACCA-3′ and 5′-TGAGTCGACCTACGTAATCGATGCCTG-3′. The resultant DNA fragments were digested with *Bam*HI and *Xho*I (Takara Bio, Shiga, Japan) and cloned into the *Bam*HI-*Xho*I site of pGEX5X-1 (GE Healthcare Japan, Tokyo, Japan) using DNA Ligation Mix (Takara Bio). The integrity of the sequence was confirmed by sequencing. The plasmids were introduced into *Escherichia coli* BL21 Codon Plus(DE3)-RIPL cells (Agilent Technologies, Santa Clara, CA, USA) by transformation. The transformed cells were added to 2 L of Luria-Bertani medium and the cells were cultured overnight at 30°C in the presence of 10 μM isopropyl-β-D-thiogalactopyranoside (Wako Chemicals, Osaka, Japan). Purification of the GST-fused FbaI was performed as previously described (Osanai et al., 2009). Immunoblotting was performed according to the method described by Osanai et al. (2014c).

### GC-MS analysis for amino acids

Equal amounts of cells (50 mL of cell culture with OD_730_ = 1.0) were harvested by rapid filtration using a previously described method (Osanai et al., 2014c). Amino acids were quantified using a GCMS-QP2010Plus apparatus (Shimadzu, Kyoto, Japan) equipped with a 10-m × 0.25-mm ZB-AAA capillary gas chromatography column. The protocol used was as described previously (Osanai et al., 2014b).

### Scanning probe microscopy

Cells grown in liquid medium were collected by centrifugation and the supernatants were discarded. The cells were resuspended in 1 mL of sterilized water and centrifuged (20,500 × *g* for 1 min) and the supernatant was again discarded. This process was repeated two more times and the cells were then resuspended in 1 mL of sterilized water. The cell suspension was spotted onto a metal plate and dried at 96°C for 1 min. The cells were observed with a scanning probe microscope (SPM-9700; Shimadzu) according to the manufacturer's instructions.

## Results

### *Synechocystis* 6803 cultivation with artificial seawater containing nitrogen and phosphorus sources

**Figure 1:**
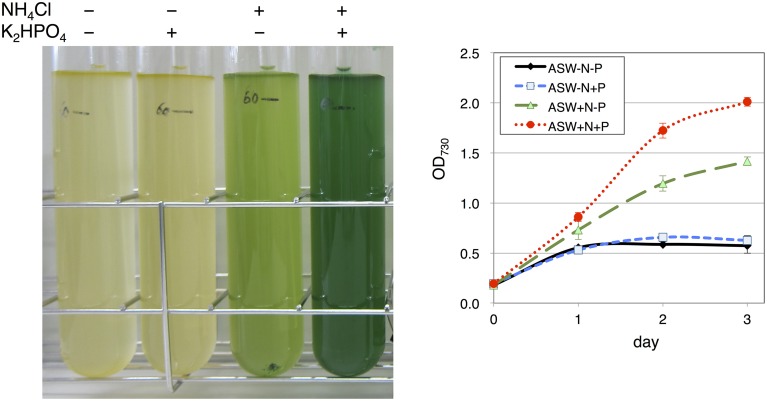
**Growth of wild-type glucose-tolerant (GT) *Synechocystis* sp. PCC 6803 cells in artificial seawater (ASW) medium**. (Left) Cell cultures were photographed after cultivation for 3 days. (Right) Growth curve of the cells in ASW with/without nitrogen and phosphorus sources. N and P designate NH_4_Cl and K_2_HPO_4_, respectively. Data represent means ± SD from three independent experiments.

Marine Art SF-1, a widely used ASW medium for the cultivation of marine bacteria and microalgae isolated from seawater (Yamane et al., 2013), was used for *Synechocystis* 6803 cultivation. The cultivation of wild-type cells was started with a the cell concentration of OD_730_ = 0.2. Cells in ASW without a nitrogen source (5 mM NH_4_Cl) hardly grew (Figure 1). Cells in the presence of the nitrogen source but in the absence of a phosphorus source (0.22 mM K_2_HPO_4_) grew partially, reaching an OD_730_ value of 1.4 for 3 days of cultivation, showing a yellow-green color (Figure 1). In the presence of both nitrogen and phosphorus sources, the cell concentration reached an OD_730_ value of 2.0 by 3 days of cultivation, showing a green color (Figure 1).

**Figure 2:**
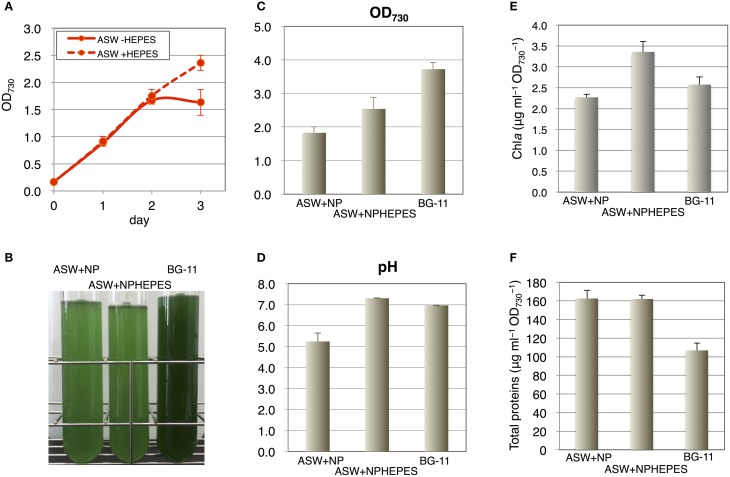
**Effect of HEPES buffer on the growth of wild-type glucose-tolerant (GT) *Synechocystis* sp. PCC 6803 cells in artificial seawater (ASW) medium. (A)** Growth curve of the cells in ASW with nitrogen and phosphorus sources with/without 20 mM HEPES buffer (pH 7.8). Data represent means ± SD from three independent experiments. **(B)** Cell cultures were photographed after cultivation for 3 days. BG-11, BG-11 medium; NPHEPES, HEPES buffer with the nitrogen and phosphorous source in the form of NH_4_Cl and K_2_HPO_4_, respectively. **(C)** OD_730_ of cell cultures after cultivation for 3 days. Data represent means ± SD from three independent experiments. **(D)** pH of cell cultures after cultivation for 3 days. Data represent means ± SD from three independent experiments. **(E)** Chlorophyll a levels of cell cultures after cultivation for 3 days. Data represent means ± SD from four independent experiments. **(F)** Intracellular soluble protein levels from the cells cultured for 3 days. Proteins were extracted by sonication and debris were removed by centrifugation. Total protein levels in the soluble fractions were quantified by BCA method. Data represent means ± SD from four independent experiments.

Because 20 mM HEPES-KOH (pH 7.8) is usually added to the medium for cyanobacterial cultivation, we next tested the effect of this buffer on cell grown in the ASW medium. Unlike the cell growth in the ASW medium without HEPES buffer, which started to decrease after 2 days, the cells grew continuously in the presence of HEPES buffer (Figure 2A). We also compared the cell growth in ASW medium with that in a synthetic medium, BG-11. The cell density reached an OD_730_ value of 3.7 in the BG-11 medium after 3 days of cultivation, but reached only to a value of ~2.5 in ASW containing nitrogen and phosphorus sources and HEPES buffer (Figures 2B,C). The pH values were around 7.0 in the BG-11 medium and in ASW with HEPES buffer, whereas the pH value was 5.0 in ASW without HEPES buffer (Figure 2D). Chlorophyll levels in the cells grown in ASW with HEPES buffer were higher than those in the other two conditions (Figure 2E). The soluble protein levels in the cells were quantified and both the protein levels of the cells grown under ASW conditions were 1.6 times of that in the cells grown in BG-11 medium (Figure 2F). Hereafter, cultivation in ASW medium indicates ASW medium containing both nitrogen and phosphorus sources.

### Glycogen content and immunoblotting

Since the ASW and BG-11 media were found to produce a difference in cell growth, we estimated the difference of primary carbon metabolism according to growth conditions. Glycogen levels were first determined using the cells cultivated for 3 days in ASW with/without HEPES buffer or in BG-11 medium (Table 1). Glycogen levels in the cells grown in the ASW medium were similar irrespective of the presence of HEPES buffer (Table 1). Glycogen levels in the cells grown in BG-11 medium was 1.7 times of that in the cells grown in ASW medium (Table 1).

**Table 1:** **Levels of glycogen after cultivation for 3 days**.

**ASW + NP**	**ASW + NPHEPES**	**BG-11**
100 ± 52.3	102.8 ± 55.0	166.5 ± 28.4

*Data represent means ± SD from six independent experiments. Levels were calibrated relative to those from glucose-tolerant Synechocystis sp. PCC 6803 cells grown in ASW without HEPES buffer (set at 100%). ASW, artificial seawater; BG-11, BG-11 medium; NP, nitrogen and phosphorus sources in the form of NH_4_Cl and K_2_HPO_4_, respectively; NPHEPES, HEPES buffer with nitrogen and phosphorous sources. Absolute values are listed in Table S2*.

**Figure 3:**
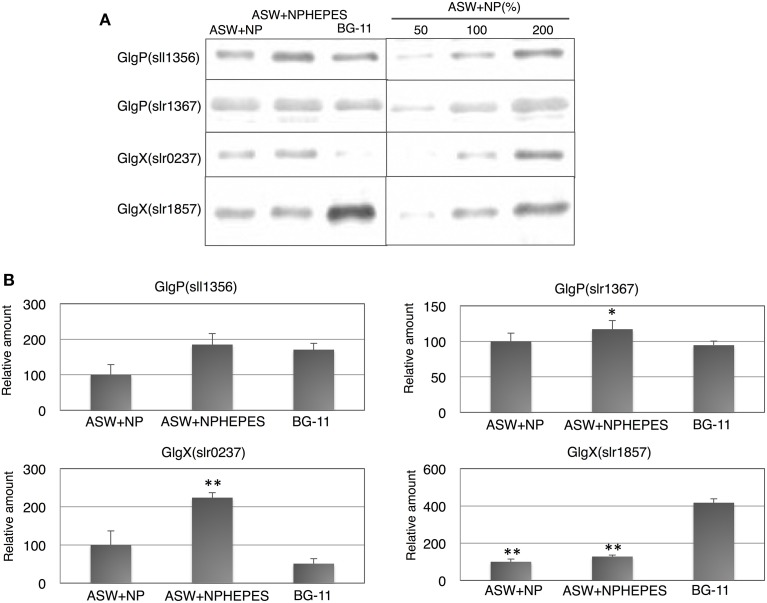
**Protein levels of four glycogen catabolic enzymes**. Immunoblotting was performed with total protein from cells grown under three different medium conditions for 3 days. **(A)** One of representative data was shown. **(B)** Data represent means ± SD from three independent experiments. Levels were calibrated relative to those of the glucose-tolerant strain grown in artificial seawater (ASW) without HEPES buffer (set at 100%). N and P designate NH_4_Cl and K_2_HPO_4_, respectively. BG-11, BG-11 medium. Asterisks (^*^ or ^**^) indicate that differences between proteins in the cells grown in ASW and BG-11 media using Student's *t*-test were statistically significant at *P* < 0.05 or *P* < 0.005, respectively.

The protein levels of glycogen catabolic enzymes, GlgP(sll1356), GlgP(slr1367), GlgX(slr0237), and GlgX(slr1857), were quantified in the cells grown for 3 days under the three medium conditions. GlgP(sll1356) protein levels in ASW with HEPES buffer and in BG-11 medium were almost 1.5–1.8 times the levels in ASW without HEPES buffer, whereas the levels of GlgP(slr1367) were similar among the three media (Figure 3). The levels of the GlgXs were fairly different among the three medium conditions. GlgX(slr0237) levels were higher in the ASW-based medium, particularly with HEPES buffer (Figure 3). On the contrary, GlgX(slr1857) level was more abundant in BG-11 medium (Figure 3).

**Figure 4:**
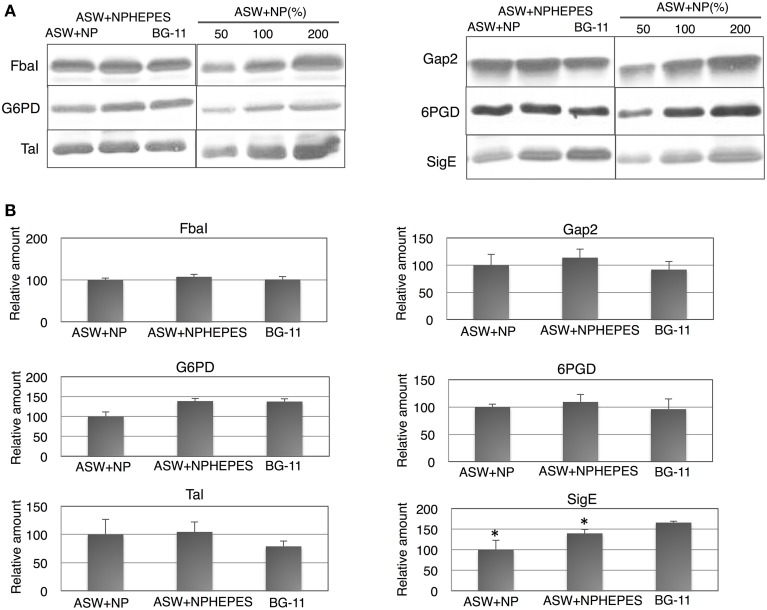
**Protein levels of for glucose catabolic enzymes and SigE**. Immunoblotting was performed with total protein from cells grown under three different medium conditions for 3 days. **(A)** One of representative data was shown. **(B)** Data represent means ± SD from three independent experiments. Levels were calibrated relative to those of the glucose-tolerant strain grown in artificial seawater (ASW) without HEPES buffer (set at 100%). N and P designate NH_4_Cl and K_2_HPO_4_, respectively. BG-11, BG-11 medium; FbaI, fructose-1,6-bisphosphate aldolase; Gap2, glyceraldehyde-3-phosphate aldolase catalyzing the anabolic reaction; G6PD, glucose-6-phosphate dehydrogenase; 6PGD, 6-phosphogluconate dehydrogenase; Tal, transaldolase; SigE, RNA polymerase group 2 sigma factor. Asterisks (^*^) indicate that differences between proteins in the cells grown in ASW and BG-11 media using Student's *t*-test were statistically significant at *P* < 0.05, respectively.

The five enzymes of glycolysis and the oxidative pentose phosphate (OPP) pathway and SigE [an RNA polymerase sigma factor activating sugar catabolism (Osanai et al., 2011)] were subsequently quantified. The protein levels of FbaI, Gap2 and 6PGD were similar among the three medium conditions tested (Figure 4). On the other hand, the G6PD levels was lower in ASW without HEPES buffer than under the other two conditions (Figure 4). The protein levels of Tal were higher in the cells grown in ASW with/without HEPES buffer than in BG-11 medium (Figure 4). SigE protein levels were higher in the order of BG-11 > ASW with HEPES buffer > ASW without HEPES buffer (Figure 4).

### Amino acid profiles in the different media

**Figure 5:**
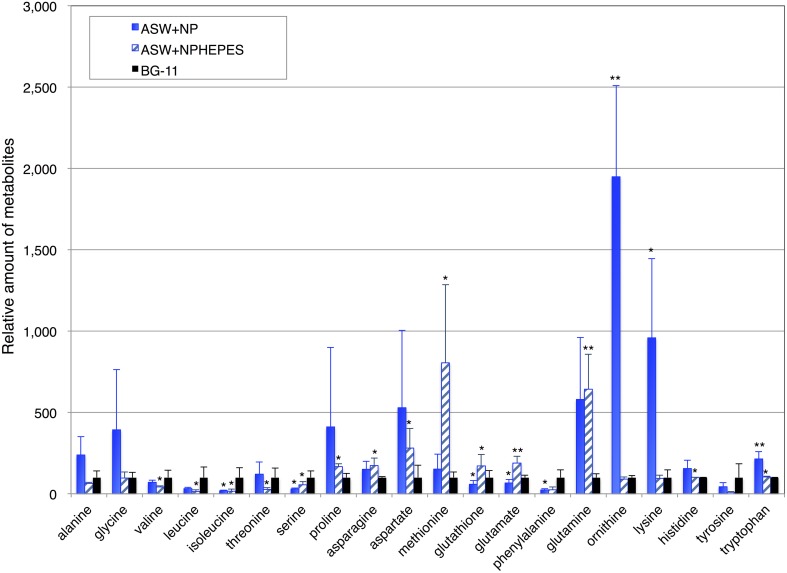
**Levels of 18 amino acids, and ornithine and glutathione**. Data represent means ± SD from five independent experiments. Levels were calibrated relative to that in cells grown in BG-11 medium (set at 100%). BG-11, BG-11 medium; ASW, artificial seawater medium; NPHEPES, HEPES buffer with the nitrogen and phosphorous source in the form of NH_4_Cl and K_2_HPO_4_, respectively. Student's *t*-test was performed and asterisks indicate statistically significant differences between BG-11 and artificial seawater with or without HEPES buffer (ASWNP or ASW NPHEPES, respectively) (^*^P < 0.05, ^**^P < 0.005).

To further clarify the effect of ASW on primary metabolism, the amino acid levels in the cells cultivated for 3 days under the three medium conditions were determined by GC-MS. The levels of proline, asparagine, aspartate, methionine, glutathione, glutamate, and glutamine in the cell grown in ASW with HEPES buffer were higher than those in the cells grown in BG-11 medium, whereas the levels of alanine, valine, leucine, isoleucine, threonine, serine, phenylalanine, and tyrosine were lower (Figure 5 and Table S1). The levels of alanine, glycine, proline, asparagine, aspartate, methionine, glutamine, ornithine, lysine, histidine, and tryptophan in cells grown in ASW without HEPES buffer were higher than those in the cells grown in BG-11 medium, whereas the levels of valine, leucine, isoleucine, serine, glutathione, phenylalanine, and tyrosine were lower (Figure 5).

### Morphological changes in artificial seawater

**Figure 6:**
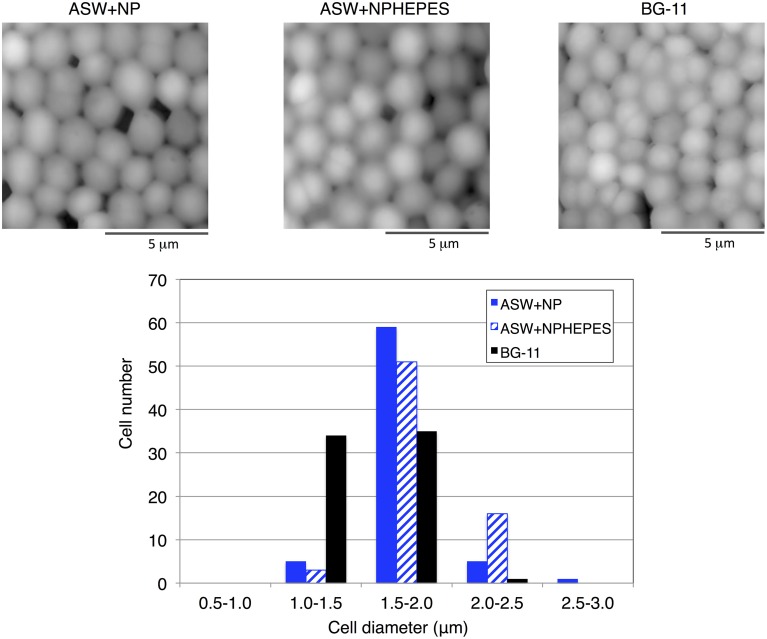
**Scanning probe micrographs of the wild-type glucose-tolerant *Synechocystis* sp. PCC 6803 cells after cultivation for 3 days under three medium conditions**. The diameter of each of 70 cells was measured.

Cell morphologies were observed by scanning probe microscopy to clarify the physiological phenotypes elicited by ASW cultivation. Whereas the cell structure and surface appearance were similar among the three medium conditions, the cell diameters were altered by ASW cultivation (Figure 6). Most of the wild-type cells in BG-11 medium were within 1.0–2.0 μm diameters (Figure 6). The majority of the cells grown in ASW with HEPES buffer had a diameter of 1.5–2.0 μm diameters but the distribution of cell diameters shifted to the 2.0–2.5 μm range (Figure 6). Many of the cells grown in ASW without HEPES buffer were also 1.5–2.0 μm in diameters, and showed a wider distribution of cell diameters from 1.0 to 3.0 μm (Figure 6).

### Growth in natural seawater

**Figure 7:**
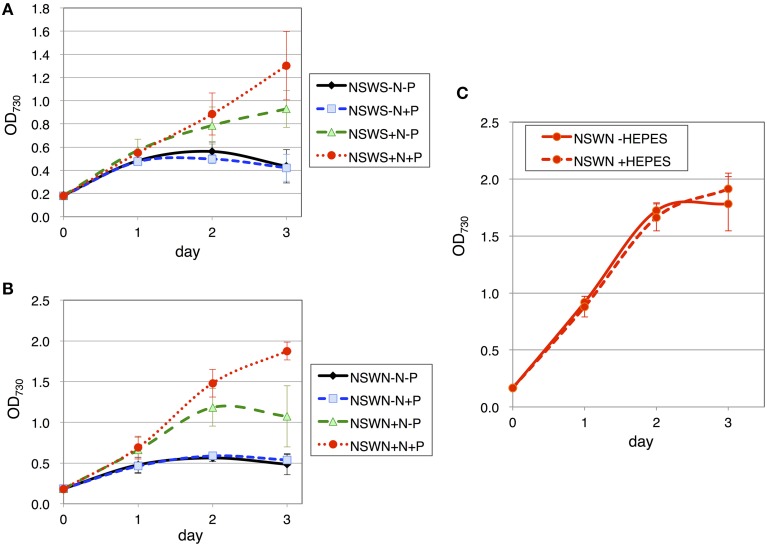
**Growth of wild-type glucose-tolerant *Synechocystis* sp. PCC 6803 cells in two natural seawater (NSW) media**. Growth curve of the cells in **(A)** NSWS (Shimano-Tennensui, seawater from Oshimo, Japan) and **(B)** NSWN (NAGEME10, seawater from the Izu Peninsula, Japan) with/without nitrogen and phosphorus sources. N and P designate NH_4_Cl and K_2_HPO_4_, respectively. Data represent means ± SD from three independent experiments. **(C)** Growth curve of the cells in NSWN supplemented with nitrogen and phosphorus sources, with/without 20 mM HEPES buffer (pH 7.8). Data represent means ± SD from three independent experiments.

Finally, the growth of the cells using natural seawater was tested. Two types of natural seawater, NSWS (from Ohshima, Japan) and NSWN (from Izu Peninsula, Japan), were purchased and tested for the effect of nitrogen and phosphorus sources. Similar to ASW, with both NSW samples, the cells grew only partially in the presence of the nitrogen sources, but they grew well when both nitrogen and phosphorus sources were present (Figures 7A,B). The cell density reached an OD_730_ value of ~1.8 in NSWN, but only reached the value of 1.3 in NSWS after 3 days of cultivation (Figures 7A,B). The addition of HEPES buffer hardly improved the growth in NSWN medium supplemented with nitrogen and phosphorus sources (Figure 7C).

## Discussion

The current results indicate the potential of seawater usage for cultivation of the freshwater cyanobacterium *Synechocystis* 6803. Although the cell growth in the seawater-based medium was still inferior to that in synthetic medium, the levels of several amino acids were highly up-regulated by ASW, demonstrating the advantage of seawater cultivation of cyanobacteria for bioproduction.

It has been revealed that *Synechocystis* 6803 cells are able to grow in an ASW medium with anaerobic digestion effluent (ADE) (Cai et al., 2013). The addition of 3% ADE to ASW gave the highest biomass productivity, but increased addition of ADE lowered the biomass and lipid productivities of *Synechocystis* 6803 (Cai et al., 2013). Other microalga such as *Nannochloropsis salina* also grew in ASW medium with ADE, but they could not grow in ASW supplemented with commercial medium containing nitrate, phosphate, and trace metals (Sheets et al., 2014). Here, we succeeded in cultivating *Synechocystis* 6803 in a seawater-based medium by adding nitrogen and phosphorus sources derived from purified chemicals (Figures 1, 7). Nitrogen was particularly indispensable for *Synechocystis* 6803 culture in both ASW and NSW media (Figures 1, 7). Furthermore, the addition of HEPES buffer improved the cell growth in ASW (Figure 2), revealing the importance of pH control for the growth of cyanobacteria in a seawater-based medium. *Synechocystis* 6803 growth in ASW was inferior to that in BG-11 medium (Figures 2B,C). Previous study revealed that *Synechocystis* cells can proliferate under 450 mM NaCl, but the growth retarded 60~70% of the cells under low salt conditions (Ferjani et al., 2003). This result is consistent with the data of Figure 2C. Our analysis demonstrated that the protein and metabolite profiles were completely different between these two medium conditions (Figures 3–5). Chlorophyll levels in the cells grown in ASW were similar to or rather higher than those in the cells grown in BG-11 (Figure 2E). These results indicate that decrease in chlorophyll levels is not the cause of poor growth in ASW. Intracellular soluble protein and glycogen levels exhibited negative correlation in our experimental conditions (Figure 2F and Table 1). NSW experiment indicates not only pH but also unknown factors limited the *Synechocystis* growth in the NSW medium (Figure 7), and further analysis is required to reveal the physiological impacts by seawater cultivation.

Immunoblotting analysis revealed that enzymes involved in the primary metabolism switch were associated with the culture conditions (Figures 3, 4), emphasizing a variety of metabolic enzymes encoded in the *Synechocystis* 6803 genome. The levels of the two GlgXs were particularly different between ASW-based and BG-11 media (Figure 3), indicating that GlgX(slr0237) may be a major isoamylase under high salt conditions. Recent omics analyses have revealed that transcript and protein levels related to primary sugar metabolism are widely altered by salt stress (Pandhal et al., 2009a; Hagemann, 2011). Transcriptome analysis has shown that the expression levels of three genes coding for proteins in sugar metabolism (viz., *pfkA*, encoding phosphofructokinase; *fbaII*, encoding fructose-1,6-bisphosphate aldolase class II; and *rpe*, encoding pentose-5-phosphate-3-epimerase) were up-regulated after salt acclimation for 24 h (Marin et al., 2004). Protein levels of glycogen phosphorylase (GlgP, slr1367), phosphoglucomutase (Pgm, sll0726), Rpe, Tal, FbaII, glucose-1-phosphate adenylyltransferase (GlgC, slr1176), and phosphoglycerate kinase (Pgk, slr0394) increased more than two-fold in cells salt-acclimated for 5 days (Fulda et al., 2006). A system biology approach based on proteomic data suggested that glycogen catabolism and glycolysis are up-regulated in salt-adapted cells, while the OPP pathway is down-regulated (Pandhal et al., 2009a,b). Thus, primary metabolism, particularly sugar metabolism, is important for salt acclimation by *Synechocystis* 6803. The protein levels of SigE, an activator of gene expression of glycogen catabolic and the OPP pathway enzymes, were higher in BG-11 than in ASW media (Figure 4). However, only G6PD protein levels were positively correlated with the SigE protein levels (Figure 4). The glycogen levels were also not correlated with SigE protein levels (Table 1). These results suggest the complicated regulatory mechanism determining the protein and metabolite levels under ASW conditions. Previous studies showed that mutation of sugar metabolism altered cell sizes and structures (Singh and Sherman, 2005; Osanai et al., 2013a), and therefore, the wide distribution of cell diameters under ASW cultivation (Figure 6) may be caused by changes in primary metabolism, aside from the salt concentration in the medium.

Amino acids are classified into six families according to their biosynthetic pathways (Umbarger, 1978). Glycine is a precursor of glycine-betaine, one of the compatible solutes accumulated under high salt conditions, and proline is a probable compatible solute in cyanobacteria (Hagemann, 2011). Proline accumulation in ASW with/without HEPES buffer may be due to the increase in salt concentration in the medium (Figure 5). The levels of four pyruvate family amino acids (isoleucine, valine, leucine, and alanine) were lower in cells grown in ASW with HEPES buffer than those in BG-11 medium, indicating that pyruvate biosynthesis may be altered by ASW cultivation. Previous metabolomic analysis showed that pyruvate in *Synechocystis* 6803 was exhausted under dark conditions (Osanai et al., 2014d), suggesting that the pyruvate pool size in this cyanobacterium is small and easily exhausted under stress conditions. Metabolomic analysis showed a negative correlation between salt stress and serine, valine, threonine, isoleucine, and tyrosine (Wang et al., 2014). That correlation study agreed well with our results, in that lower levels of serine, valine, threonine, isoleucine, and tyrosine were found in the cells grown in ASW with HEPES buffer than those in BG-11 medium (Figure 5). Alanine and glutamine were found to correlate positively with salt stress (Wang et al., 2014), a finding consistent with our results (Figure 5). Therefore, the differences in amino acid profiles among three medium conditions can be partly explained by the salt conditions. However, there were large differences in amino acid profiles between ASW with and ASW without HEPES buffer, suggesting that other factors, such as pH and cell growth, may affect the amino acid profiles. Poly-lysine production in *Streptomyces* sp. M-Z18 increased by acidic pH shock (Ren et al., 2015), and thus, decrease in pH may be cause of increase in lysine in the cells grown in ASW without HEPES buffer. The values of standard deviation of amino acid levels in the cells cultivated in ASW without HEPES were larger than the other two conditions (Figure 5), which may reflect less growth in ASW without HEPES buffer after 3-day cultivation. Future study to reveal the cause–result effect in seawater-based media will explain the changes in primary metabolism and growth of cyanobacteria.

### Conflict of interest statement

The authors declare that the research was conducted in the absence of any commercial or financial relationships that could be construed as a potential conflict of interest.
